# The association between the sodium-potassium ratio and ICU mortality in cardiac arrest patients: an analysis of the eICU database

**DOI:** 10.3389/fmed.2026.1855533

**Published:** 2026-06-05

**Authors:** Yuyuan Wang, Xin Yuan, Dongxia Wang, Mingli Li, Ruihua Wang

**Affiliations:** 1 Department of Cardiology, Changzhi People’s Hospital, The Affiliated Hospital of Changzhi Medical College, Changzhi, Shanxi, China; 2 Department of Critical Care Medicine, Changzhi People’s Hospital, The Affiliated Hospital of Changzhi Medical College, Changzhi, Shanxi, China; 3 Department of Cardiology, Shanxi Provincial People’s Hospital, Taiyuan, Shanxi, China

**Keywords:** cardiac arrest, eICU database, ICU mortality, potassium, sodium

## Abstract

**Background:**

Electrolyte imbalance, particularly abnormalities in potassium and sodium homeostasis, represents an important and potentially modifiable risk factor for in-hospital cardiac arrest. Severe disturbances in potassium or sodium levels can precipitate fatal arrhythmias and are associated with poor outcomes following resuscitation. Timely correction of these electrolyte disorders may therefore reduce mortality in patients with cardiac arrest. Although the urine sodium-potassium (Na^+^/K^+^) ratio has been shown to be associated with prognosis in various cardiovascular diseases, the prognostic value of the serum Na^+^/K^+^ ratio in predicting intensive care unit (ICU) mortality among patients with cardiac arrest remains incompletely understood. This study aims to investigate the association between the serum Na^+^/K^+^ ratio in patients following cardiac arrest and the risk of ICU mortality.

**Methods:**

We screened 4,085 ICU-admitted cardiac arrest patients from the eICU-CRD databases, stratified by Na^+^/K^+^ ratio tertiles. Endpoints was ICU mortality. Analyses used logistic regression, restricted cubic splines, and subgroup analyses.

**Results:**

A total of 4,085 patients met the inclusion criteria, including 2,392 males, with a mean age of 63.7 years. A U-shaped association was observed between the Na^+^/K^+^ ratio and ICU mortality. In the fully adjusted model, a higher Na^+^/K^+^ ratio was significantly associated with reduced ICU mortality (adjusted OR = 0.75; 95% CI: 0.64–0.89). The prognostic value of the Na^+^/K^+^ ratio for ICU mortality remained consistent across all subgroups, with no significant interactions observed.

**Conclusion:**

To summarize, we identified a marked U-shaped relationship between the serum Na^+^/K^+^ ratio and ICU mortality among cardiac arrest patients. Routine assessment of this metric could facilitate risk stratification and inform clinical management.

## 1 Introduction

Cardiac arrest (CA) remains a major global public health burden. In the United States, the annual incidence of emergency medical services (EMS)-treated out-of-hospital cardiac arrest (OHCA) is approximately 356,000 (83 per 100,000 population), while the estimated annual number of in-hospital cardiac arrests (IHCA) reaches 292,000. Globally, the average incidence of adult CA is approximately 55 per 100,000 person-years, with considerable regional disparities in survival outcomes. This high incidence and persistently poor prognosis underscore an urgent need to identify novel prognostic biomarkers to improve risk stratification and guide clinical decision-making in CA patients (1). The current research landscape reveals a notable disparity between OHCA and IHCA: while OHCA has been the primary focus of randomized controlled trials (RCTs) and systematic reviews, IHCA has received comparatively less attention, resulting in substantial gaps in evidence-based management strategies for patients who experience arrest in clinical settings (2). Furthermore, the field continues to face several critical challenges, including limited research funding, difficulties in data acquisition attributable to the time-sensitive nature of CA events, and the need for more robust multicenter registries to capture comprehensive patient outcomes and facilitate the translation of research findings into clinical practice (3, 4). In addition, systematic evaluation of CA survivors may identify the underlying etiology of unexplained cardiac arrest, which is essential not only for preventing recurrence but also for guiding the long-term management of both patients and their family members (5).

The sodium-to-potassium (Na^+^/K^+^) ratio has emerged as a more robust predictor of cardiovascular disease (CVD) risk than either sodium or potassium intake alone. Accumulating epidemiological evidence consistently demonstrates a dose-response relationship, wherein higher Na^+^/K^+^ intake or excretion ratios are associated with a significantly increased incidence of major cardiovascular events, including stroke, coronary heart disease, and heart failure (6–8). Notably, a longitudinal follow-up study from the Trials of Hypertension Prevention (TOHP) cohort reported that participants in the highest quartile of urinary Na^+^/K^+^ ratio exhibited a 1.5-fold greater risk of subsequent CVD events compared with those in the lowest quartile, independent of traditional cardiovascular risk factors (6). Furthermore, a pooled analysis of international cohorts corroborated these findings, demonstrating that each one-unit increase in the Na^+^/K^+^ intake ratio was associated with a 24% increase in cardiovascular risk (9). Collectively, these data underscore the clinical utility of the Na^+^/K^+^ ratio as a composite biomarker for CVD risk stratification and highlight the potential benefits of dietary interventions aimed at reducing sodium intake while increasing potassium consumption to mitigate cardiovascular morbidity and mortality (7, 10).

Current evidence indicates that elevated serum potassium levels at the time of admission are persistently associated with worse neurological outcomes and lower survival rates following out-of-hospital cardiac arrest (11, 12). In contrast, the Na^+^/K^+^ intake ratio has been primarily employed as an indicator of overall cardiovascular mortality, rather than as a direct predictor of cardiac arrest prognosis (13). However, the role of the serum Na^+^/K^+^ ratio in determining outcomes following cardiac arrest remains insufficiently elucidated. To the best of our knowledge, the present study is the first to investigate the association between the serum Na^+^/K^+^ ratio and clinical outcomes in patients with cardiac arrest.

## 2 Materials and methods

### 2.1 Data source

Study participants were drawn from the eICU Collaborative Research Database (eICU-CRD) (14), a large, publicly available multicenter repository designed to support critical care research. The database comprises de-identified clinical records from more than 200,000 ICU admissions across 208 hospitals in the United States during the period from 2014 to 2015. Comprehensive data are available for each admission, including patient demographic characteristics, vital signs, laboratory findings, clinical diagnoses, and therapeutic interventions, all derived from bedside monitoring systems and clinical documentation (14). The eICU-CRD is maintained by the Philips eICU Research Institute and is accessible to qualified investigators upon completion of required ethics training and execution of a formal data use agreement. Data extraction for the present study was performed by an authorized investigator who had been granted access to the database.

### 2.2 Study design

**FIGURE 1:**
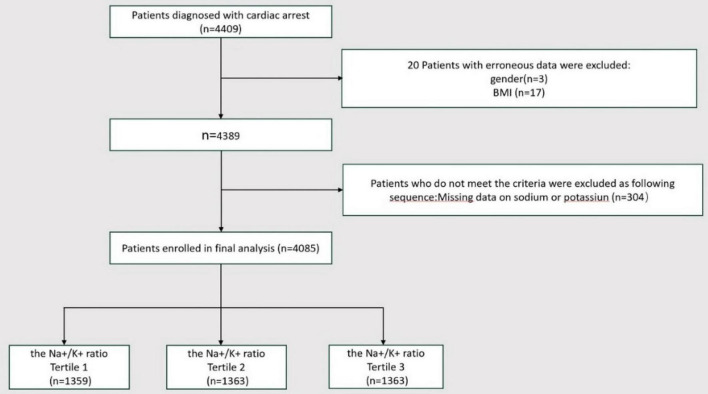
Flowchart of patient screening.

This study employed a retrospective, observational, multicenter cohort design. Among a total of 200,859 ICU admission records in the eICU-CRD, we initially identified 4,409 patients with in-hospital cardiac arrest based on the International Classification of Diseases, Ninth Revision (ICD-9) code 4,275, after excluding individuals under 18 years of age. Application of additional exclusion criteria—including patients with physiologically implausible values (*n* = 20) and those with incomplete clinical data (*n* = 304)—yielded a final analytical cohort of 4,085 patients (Figure 1). Given that all data in the database have been de-identified to protect patient privacy, neither informed consent nor institutional review board approval was required for this study.

### 2.3 Variable extraction

Data were extracted from the eICU-CRD across three domains: (1) baseline demographic characteristics, including sex, age, race (Caucasian, African American, Hispanic, Asian, Native American, and other/unknown), body weight, and height; (2) vital signs, including heart rate, respiratory rate, and body temperature; and (3) laboratory biomarkers, including blood urea nitrogen (BUN), calcium, chloride, creatinine, hematocrit (HCT), hemoglobin (HGB), magnesium, mean corpuscular hemoglobin (MCH), mean corpuscular hemoglobin concentration (MCHC), mean corpuscular volume (MCV), potassium, red blood cell (RBC) count, sodium, white blood cell (WBC) count, and platelet count. All variables were extracted from the first recorded values obtained within 24 h of ICU admission. Variables with a missing rate ≥ 20% were excluded from further analysis, while continuous variables with missing rates < 20% were imputed using multiple imputation methods. All statistical analyses were performed using Free Statistics software, version 2.4.

### 2.4 Groups and outcomes

The exposure factor, Serum Na^+^/K^+^ ratio, was determined by dividing Sodium (mmol/L) by potassium (mmol/L). Among the 208 hospitals that provided data to the eICU-CRD database, our final analysis sample group included patients from 165 hospitals (accounting for 79%) who met the inclusion criteria during the study period (2014–2015). This sample group consisted of 4,085 adult patients, with a total of 1,586 cases of intensive care unit deaths (mortality rate: 38.8%). Based on the tertiles of the Na^+^/K^+^ ratio, they were divided into three groups: T1 (≤30.91, *n* = 1,359), T2 (30.93–36.67, *n* = 1,363), and T3 (≥36.75, *n* = 1,363).The primary outcome was ICU mortality, ICU mortality was defined as death occurring during the ICU stay.

**TABLE 1:** Comparisons of the baseline characteristics categorized by the Na^+^/K^+^ ratio index.

Variables	Total (*n* = 4085)	Na^+^/K^+^ ratio tertile 1, (*n* = 1359)	Na^+^/K^+^ ratio tertile 2, (*n* = 1363)	Na^+^/K^+^ ratio tertile 3, (*n* = 1363)	*P*-value
Patent characteristics					
Gender, n (%)					<0.001
Male	2392 (58.6)	835 (61.4)	840 (61.6)	717 (52.6)	
Female	1693 (41.4)	524 (38.6)	523 (38.4)	646 (47.4)	
Age, (years)	63.7 ± 15.6	64.7 ± 14.8	63.8 ± 15.8	62.7 ± 16.0	0.005
Race, *n* (%)					0.479
Caucasian	3,026 (74.1)	1,005 (74)	1,031 (75.6)	990 (72.6)	
African American	578 (14.1)	196 (14.4)	183 (13.4)	199 (14.6)	
Hispanic	149 (3.6)	51 (3.8)	41 (3)	57 (4.2)	
Asian	71 (1.7)	23 (1.7)	19 (1.4)	29 (2.1)	
Native American	30 (0.7)	12 (0.9)	6 (0.4)	12 (0.9)	
Other/unknown	231 (5.7)	72 (5.3)	83 (6.1)	76 (5.6)	
Diabetes, *n* (%)					0.514
No	3,407 (83.4)	1,131 (83.2)	1,149 (84.3)	1,127 (82.7)	
Yes	678 (16.6)	228 (16.8)	214 (15.7)	236 (17.3)	
Hypertension, *n* (%)					0.25
No	1,907 (46.7)	654 (48.1)	640 (47)	613 (45)	
Yes	2,178 (53.3)	705 (51.9)	723 (53)	750 (55)	
Vital signs					
BMI, *n* (%)	29.8 ± 8.3	30.7 ± 8.6	29.5 ± 8.0	29.1 ± 8.2	<0.001
Heart rate, (beats/min)	90.5 ± 22.7	89.8 ± 23.0	90.4 ± 22.0	91.3 ± 23.2	0.216
Respiratory rate, (times/min)	20.5 ± 6.4	20.9 ± 6.5	20.3 ± 6.1	20.4 ± 6.7	0.015
Temperature, (°C)	35.9 ± 1.5	36.0 ± 1.4	36.1 ± 1.4	35.8 ± 1.6	<0.001
Lab values					
Albumin, (g/L)	2.8 ± 0.6	2.8 ± 0.6	2.9 ± 0.6	2.8 ± 0.6	<0.001
BUN, (mg/dl)	24.0 (16.0, 38.0)	35.0 (22.0, 51.0)	21.0 (15.0, 33.0)	20.0 (14.0, 30.0)	<0.001
Creatinine, (mg/dl)	1.4 (0.9, 2.2)	1.9 (1.3, 3.2)	1.2 (0.9, 1.9)	1.2 (0.8, 1.7)	<0.001
Sodium, (mmol/L)	138.9 ± 5.6	136.8 ± 5.7	139.0 ± 4.8	141.0 ± 5.4	<0.001
Potassium, (mmol/L)	4.3 ± 0.9	5.3 ± 0.8	4.1 ± 0.2	3.4 ± 0.4	<0.001
Sodium/potassium ratio, (index)	34.3 ± 7.8	26.4 ± 3.4	33.8 ± 1.6	42.6 ± 6.2	<0.001
Calcium, (mmol/L)	8.1 ± 1.0	8.3 ± 1.2	8.2 ± 0.9	8.0 ± 1.0	<0.001
Magnesium, (mmol/L)	2.0 ± 0.5	2.1 ± 0.5	2.0 ± 0.5	1.9 ± 0.4	<0.001
Chloride, (mmol/L)	104.2 ± 6.7	102.2 ± 6.9	104.7 ± 6.1	105.6 ± 6.5	<0.001
HCT, (%)	35.0 ± 7.6	34.0 ± 7.7	35.4 ± 7.4	35.6 ± 7.5	<0.001
HGB, (g/L)	11.5 ± 2.6	11.0 ± 2.6	11.7 ± 2.6	11.7 ± 2.6	<0.001
Platelet count, (10^9^/L)	204.0 ± 88.2	205.3 ± 91.9	204.1 ± 83.4	202.5 ± 89.2	0.698
RBC, (10^12^/L)	3.9 ± 0.9	3.7 ± 0.9	3.9 ± 0.8	3.9 ± 0.9	<0.001
WBC, (10^9^/L)	13.9 (9.7, 19.0)	14.6 (10.2, 19.9)	13.6 (9.5, 18.4)	13.6 (9.4, 19.0)	<0.001
MCH, (pg)	29.7 ± 2.6	29.5 ± 2.7	29.9 ± 2.4	29.8 ± 2.6	<0.001
MCHC, (g/L)	32.7 ± 1.5	32.3 ± 1.6	32.8 ± 1.4	32.9 ± 1.5	<0.001
MCV, (fL)	91.1 ± 6.9	91.5 ± 7.3	91.1 ± 6.5	90.8 ± 6.9	0.02
Rdw, (%)	15.4 ± 2.4	15.9 ± 2.5	15.2 ± 2.3	15.2 ± 2.2	<0.001
Patient outcomes					
ICU mortality, *n* (%)					<0.001
Alive	2,499 (61.2)	756 (55.6)	908 (66.6)	835 (61.3)	
Expired	1,586 (38.8)	603 (44.4)	455 (33.4)	528 (38.7)	
Unitlosday, (day)	3.1 (1.4, 6.2)	2.9 (1.1, 6.0)	3.0 (1.4, 6.0)	3.3 (1.7, 6.7)	<0.001

BMI, body mass index; RDW, red blood cell distribution width; BUN, blood urea nitrogen; HCT, hematocrit; HGB, hemoglobin; MCH, mean corpuscular hemoglobin; MCHC, mean corpusular hemoglobin concerntration; MCV, mean corpuscular volume; WBC, white blood cell; RBC, red blood cell.

### 2.5 Statistical analysis

Continuous data with a normal distribution are provided as mean ± SD and analyzed using one-way ANOVA. Data with a skewed distribution are presented as median with quartile spacing [M (Q1, Q3)] and analyzed using the Kruskal-Wallis test. Categorical variables are reported as *n* (%) and analyzed using the chisquare test (or Fisher’s exact test).

Univariate and multivariate logistic proportional odds regression models were applied to evaluate the association between the Na^+^/K^+^ ratio and ICU all-cause mortality, with odds ratios (ORs) and 95% confidence intervals (CIs) reported. We conducted covariate screening using Free Statistics software and combined with clinical expertise to finally identify the covariates (Supplementary Material 1: Supplementary Table 1). Model 1 was adjusted for race, gender, age and heart rate. Model 2 was further adjusted for race, gender, age and heart rate, albumin, calcium, chloride, creatinine, magnesium and WBC. We conducted subgroup analyses because critically ill patients exhibit substantial clinical heterogeneity in organ function, comorbidities, and disease severity. The association between the Na^+^/K^+^ ratio and cardiac arrest may vary across different patient populations. Through subgroup analyses, we aimed to explore potential effect modification and identify high-risk subgroups. Subgroup analyses were performed stratified by demographic characteristics, including gender, age, BMI, hypertension, and diabetes.

We acknowledge that patients treated within the same hospital may share unmeasured characteristics (e.g., institutional protocols, staffing patterns, resource availability) that could induce intra-hospital correlation in outcomes. We addressed this through two complementary approaches: (1) Primary analysis with clustered bootstrap standard errors: We re-estimated all Cox proportional hazard models using a cluster-resampling bootstrap approach, where entire hospitals (rather than individual patients) were resampled with replacement. This preserves the intra-hospital correlation structure and provides valid statistical inference. We performed 300 bootstrap replications and derived percentile-based 95% confidence intervals. (2) Sensitivity comparison with naive standard errors: For transparency, we also report results using conventional (naive) standard errors that ignore clustering, allowing readers to assess the magnitude of clustering effects on our estimates (Supplementary Material 1: Supplementary Table 2). All analyses were conducted using Free Statistics software, version 2.4 (15), and R^1^ (The R Foundation for Statistical Computing).

## 3 Results

### 3.1 Patient and hospital characteristics

To address intra-hospital correlation within this multicenter cohort, we compared naive and hospital-clustered bootstrap standard errors. Point estimates were identical between the two approaches, with clustered confidence intervals only marginally widened and no changes in statistical significance. This confirms that institutional heterogeneity exerted minimal bias, verifying the robustness of our primary findings (Supplementary Material 1: Supplementary Table 2).

This study comprised a total of 4,085 patients with cardiac arrest. The baseline characteristics of the patients are summarized in Table 1. Patients were stratified into three groups according to tertiles of the serum Na^+^/K^+^ ratio: 1,359 in the lowest tertile group (≤30.91), 1,363 in the middle tertile group (30.93–36.67), and 1,363 in the highest tertile group (≥36.75).

Compared with patients in the lowest tertile of the serum Na^+^/K^+^ ratio, those in the highest tertile were significantly younger and had a lower body mass index (BMI) at admission. They also exhibited a higher heart rate, lower respiratory rate, and lower body temperature. Laboratory findings revealed significantly lower levels of albumin, RDW, BUN, calcium, MCH, MCHC, MCV, platelet count, and WBC, alongside significantly higher levels of HCT, HGB, and RBC. Notably, patients in the highest tertile had significantly higher ICU mortality rates and longer ICU lengths of stay compared with those in the lowest tertile (Table 1).

### 3.2 ICU mortality of cardiac arrest is associated with serum Na^+^/K^+^ ratio

Univariate analysis results are presented in Table 2. The following variables were significantly associated with ICU mortality: age, sex, albumin, RDW, respiratory rate, temperature, heart rate, BUN, calcium, creatinine, HGB, MCHC, MCV, platelet count, potassium, sodium, Na^+^/K^+^ ratio, RBC, and WBC.

**TABLE 2:** Association of covariates and intensive care unit (ICU) mortality.

Variable	OR (95% CI)	*P*-value
Patent characteristics		
Gender, n (%)		
Female	1 (reference)	
Male	1.19 (1.05–1.36)	0.007
Age, (years)	1.01 (1–1.01)	0.005
Race, n (%)		
Caucasian	1 (reference)	–
African American	0.9 (0.75–1.09)	0.281
Hispanic	0.76 (0.54–1.08)	0.132
Asian	1.6 (1–2.57)	0.049
Native American	1.56 (0.76–3.2)	0.226
Other/unknown	1.03 (0.79–1.36)	0.818
Diabetes, n (%)		
	1 (reference)	
	0.97 (0.82–1.15)	0.715
Hypertension, n (%)		
	1 (reference)	
	1.08 (0.96–1.23)	0.212
Vital signs		
BMI, *n* (%)	1 (0.99–1.01)	0.787
Heartrate, (beats/min)	1.01 (1–1.01)	<0.001
Respiratoryrate, (beats/min)	1.03 (1.02–1.04)	<0.001
Temperature, (°C)	0.74 (0.71–0.78)	<0.001
Lab values		
Albumin, (g/L)	0.53 (0.48–0.59)	<0.001
Creatinine, (mg/dl)	1.11 (1.07–1.15)	<0.001
BUN, (mg/dl)	1.01 (1.01–1.02)	<0.001
Calcium, (mmol/L)	0.86 (0.81–0.92)	<0.001
Magnesium, (mmol/L)	1.29 (1.14–1.47)	<0.001
Sodium-potassium ratio	0.99 (0.98–1)	0.026
Sodium, (mmol/L)	1.03 (1.02–1.05)	<0.001
Potassium, (mmol/L)	1.19 (1.11–1.27)	<0.001
Chloride, (mmol/L)	1 (0.99–1.01)	0.732
HCT, (%)	0.99 (0.99–1)	0.163
HGB, (g/L)	0.96 (0.94–0.98)	0.001
MCH, (pg)	0.98 (0.96–1)	0.099
MCHC, (g/L)	0.81 (0.78–0.84)	<0.001
MCV, (fL)	1.02 (1.01–1.03)	<0.001
Rdw, (fL)	1.13 (1.1–1.16)	<0.001
Platelet count, (10^9^/L)	1 (1–1)	<0.001
RBC, (10^12^/L)	0.92 (0.85–0.98)	0.018
WBC, (10^9^/L)	1.03 (1.02–1.04)	<0.001

BMI, body mass index; RDW, red blood cell distribution width; BUN, blood urea nitrogen; HCT, hematocrit; HGB, hemoglobin; MCH, mean corpuscular hemoglobin; MCHC, mean corpusular hemoglobin concerntration; MCV, mean corpuscular volume; WBC, white blood cell; RBC, red blood cell.

Logistic proportional hazards regression analysis results are presented in Table 3. When the Na^+^/K^+^ ratio was analyzed as a categorical variable (by tertiles), patients in the middle tertile (T2) had significantly lower ICU mortality compared with those in the lowest tertile (T1) in both the unadjusted model (OR = 0.63; 95% CI: 0.54–0.73) and Model 1 adjusted for race, gender, age and heart rate (OR = 0.63; 95% CI: 0.54–0.73). In the fully adjusted model further controlling for albumin, calcium, chloride, creatinine, magnesium and WBC (Model 2), the inverse association was most pronounced in the higher tertile (T2), with a 25% reduction in ICU mortality compared with T1 (OR = 0.75; 95% CI: 0.64–0.89).

**TABLE 3:** Odds ratios (95% confidence intervals) of intensive care unit (ICU) mortality and different Na^+^/K^+^ ratio in different models.

Outcomes	Group	Non-adjusted		Model 1		Model 2	
		OR (95% CI)	*P*	OR (95% CI)	*P*	OR (95% CI)	*P*
ICU mortality	Na^+^/K^+^ tertile 1	1 (ref)	–	1 (ref)	–	1 (ref)	–
	Na^+^/K^+^ tertile 2	0.63 (0.54∼0.73)	<0.001	0.63 (0.54∼0.73)	<0.001	0.75(0.64∼0.89)	0.001
	Na^+^/K^+^ tertile 3	0.79 (0.68∼0.92)	0.003	0.78(0.67∼0.91)	0.002	0.91 (0.77∼1.08)	0.263

Model 1 was adjusted for race, gender, age and heart rate. Model 2 was additionally adjusted for albumin, calcium, chloride, creatinine, magnesium and WBC.

**FIGURE 2:**
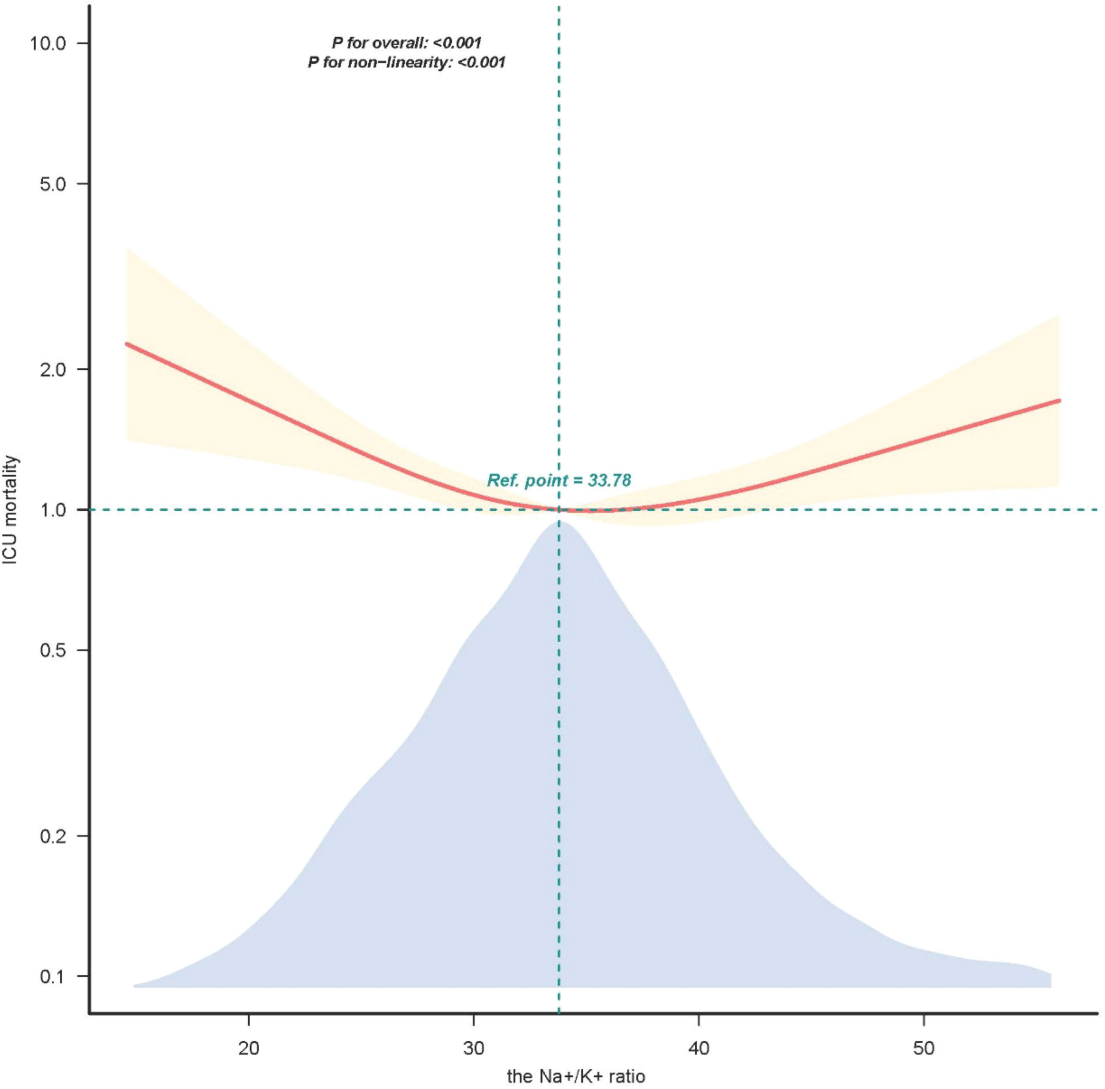
Restricted cubic spline curves for intensive care unit (ICU) mortality by Na^+^/K^+^ ratio in all participants after covariate adjustment.

Restricted cubic spline (RCS) analysis revealed a significant U-shaped relationship between the serum Na^+^/K^+^ ratio and ICU mortality (non-linear, *p* < 0.001) (Figure 2). In the threshold analysis, the OR of ICU mortality was 0.964 (95% CI: 0.941–0.988) in participants with Na^+^/K^+^ ratio less than 33.836. Beyond the 33.836 threshold, higher Na^+^/K^+^ ratio was associated with a 2.6% increased odds of ICU mortality per one-unit (OR = 1.026, 95% CI: 1.006–1.0646) (Table 4).

**TABLE 4:** Threshold effect analysis of Na^+^/K^+^ ratio on intensive care unit (ICU) mortality.

Threshold of Na^+^/K^+^ ratio	OR	95% CI	*P-*value
<33.836	0.964	0.941–0.988	0.0034
≥33.836	1.026	1.006–1.046	0.0094

Adjusted for all covariates in Table 3.

### 3.3 Subgroup analyses and sensitivity analyses

**FIGURE 3:**
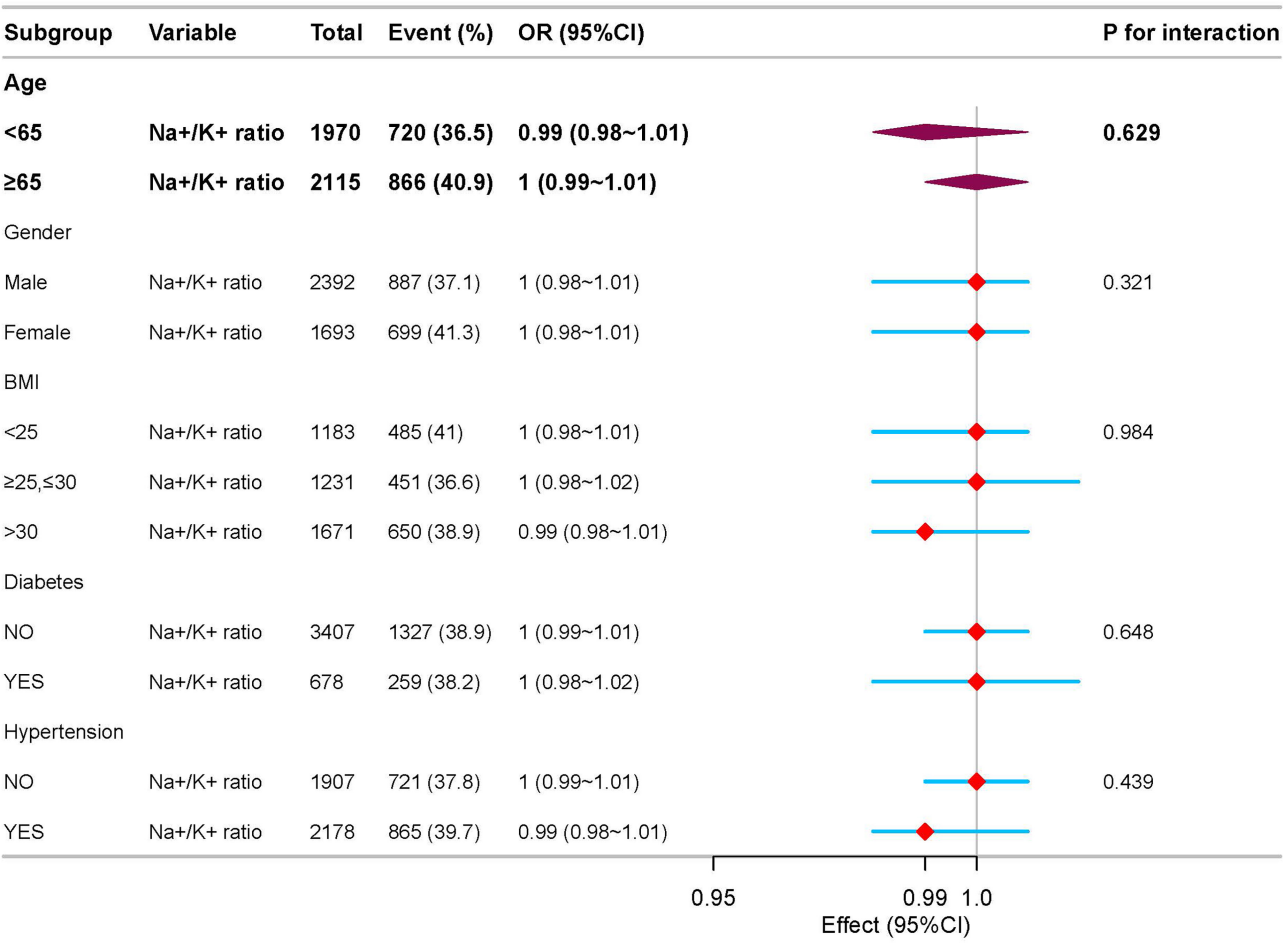
Forest plots of odds ratios for the in- intensive care unit (ICU) mortality in different subgroup. OR, odds ratio; CI, confidence interval; BMI, body mass index.

With respect to the primary outcome, the risk stratification value of the serum Na^+^/K^+^ ratio was evaluated across prespecified subgroups defined by age, sex, BMI, hypertension, and diabetes (Figure 3). No significant interactions were observed between the serum Na^+^/K^+^ ratio and any of the subgroups (all P for interaction > 0.05). Furthermore, as a sensitivity analysis, we also conducted a proportional analysis and Cox multivariate regression analysis. We found that none of these factors were likely to affect our results (Supplementary Material 2: Supplementary Figure 1, Supplementary Tables 1, 2).

## 4 Discussion

Cardiac arrest is one of the leading causes of death worldwide, characterized by the sudden cessation of cardiac mechanical activity, resulting in circulatory failure and interruption of blood supply to vital organs. Despite continuous advances in cardiopulmonary resuscitation techniques and emergency response systems, the overall survival rate following cardiac arrest remains low. The survival-to-discharge rate is approximately 8%–12% for out-of-hospital cardiac arrest and approximately 25% for in-hospital cardiac arrest (16, 17). Even among patients who achieve successful resuscitation, many may still suffer from serious neurological sequelae, including hypoxic-ischemic encephalopathy and cognitive dysfunction (18). Therefore, early identification of high-risk patients and timely intervention are crucial for improving clinical outcomes.

Sodium and potassium are the key electrolytes that maintain normal cardiac electrophysiology. Sodium ions primarily mediate the rapid depolarization phase (phase 0) of the cardiac action potential, and their inward influx triggers myocardial contraction(19). Hypernatremia can lead to expansion of extracellular fluid volume and hypertension, and has been associated with an increased risk of cardiovascular events (20). In contrast, hyponatremia may cause cellular edema and neurological dysfunction, and is strongly linked to poor prognosis in patients with heart failure (21). Potassium ions play a central role in myocardial repolarization. The concentration gradient of potassium across the cell membrane determines the resting membrane potential and directly influences myocardial excitability and conductivity (22). Both hyperkalemia and hypokalemia have been reported to be associated with an increased risk of in-hospital cardiac arrest (23). Hypokalemia can prolong action potential duration and increase the risk of early afterdepolarization, thereby predisposing to arrhythmias, including torsades de pointes and ventricular fibrillation (24). Conversely, hyperkalemia reduces myocardial excitability, may cause conduction block, and can precipitate cardiac arrest (25). Notably, even within the normal reference range, minor fluctuations in serum potassium levels can significantly affect cardiac rhythm stability (26).

The Na^+^/K^+^ ratio, as a composite indicator, provides a comprehensive assessment of systemic electrolyte balance. Previous studies have demonstrated that the Na^+^/K^+^ ratio is significantly associated with hypertension, cardiovascular diseases, and all-cause mortality (13). The underlying pathophysiological mechanisms may involve several aspects. First, an elevated Na^+^/K^+^ ratio may reflect relative hypokalemia. In clinical practice, serum sodium levels tend to remain relatively stable, whereas serum potassium levels are more susceptible to modulation by dietary intake, medications, and renal function. A high Na^+^/K^+^ ratio often indicates relatively low serum potassium, which may increase the risk of arrhythmia and cardiac arrest (27). Second, the Na^+^/K^+^ ratio may be associated with activation of the renin–angiotensin–aldosterone system (RAAS). Aldosterone promotes sodium retention and potassium excretion, leading to an elevated Na^+^/K^+^ ratio. Overactivation of the RAAS not only contributes to myocardial fibrosis and ventricular remodeling but also increases arrhythmia susceptibility through electrolyte disturbances (28). Third, the Na^+^/K^+^ ratio may serve as an indicator of renal capacity to regulate electrolyte homeostasis. Patients with chronic kidney disease commonly present with electrolyte imbalances, and an abnormal Na^+^/K^+^ ratio may reflect impaired renal function, which is itself an independent risk factor for cardiac arrest (29).

To the best of our knowledge, this is the first study to demonstrate that a higher serum Na^+^/K^+^ ratio is significantly and independently associated with reduced ICU mortality in patients with cardiac arrest, even after comprehensive multivariable adjustment. RCS analysis revealed a non-linear association between the serum Na^+^/K^+^ ratio and ICU mortality, with the OR curve demonstrating a U-shaped pattern (*P* < 0.001), and both low and high Na^+^/K^+^ ratios were associated with increased mortality risk. The threshold analysis indicated that the lowest risk point occurred around approximately 33.836. This optimal range likely reflects a physiological equilibrium between sodium retention and potassium depletion, which is essential for maintaining intracellular homeostasis. These findings underscore the clinical value of monitoring this ratio to identify patients at elevated risk.

These findings hold significant clinical implications. First, the serum Na^+^/K^+^ ratio is a simple and readily obtainable marker that can be assessed at the bedside, facilitating the early identification of high-risk patients. Second, the observed non-linear relationship suggests the existence of a critical threshold, below which mortality risk increases substantially, thereby providing a quantifiable reference point for clinical decision-making. Third, no significant interactions were detected in any of the subgroup analyses, suggesting that the risk-stratification value of the serum Na^+^/K^+^ ratio is broadly applicable across diverse patient populations.

Several limitations of the present study should be acknowledged. First, although we adjusted for potential confounders and performed subgroup analyses, the retrospective design inherently carries the risk of unmeasured confounding and selection bias. Prospective cohort studies are warranted to validate these findings. Second, we only evaluated the initial serum Na^+^/K^+^ ratio within 24 h of ICU admission without continuous or dynamic monitoring; however, this value may fluctuate with clinical progression and during the post-cardiac arrest hospitalization. Third, some statistically significant differences in baseline characteristics may lack clinical significance, as they were derived from multiple non-hypothesis-driven comparisons within a large sample, such as body temperature and respiratory rate. Notable baseline differences were also observed between included and excluded patients, which suggest potential selection bias and may further restrict the external generalizability of our findings. Fourth, due to the inherent limitations of the eICU database, inter-hospital ICU transfers were not trackable, and patients with multiple ICU admissions were included only once using the first admission. Fifth, Patients who survived to hospital discharge after ICU transfer but subsequently died were not included in this outcome. Finally, the temporal uncertainty regarding whether cardiac arrest occurred before or after the serum Na/K ratio measurement, along with important time-varying confounders not captured by the eICU database—including the presence and timing of dialysis, fluid interventions such as large-volume saline boluses before measurement, and electrolyte repletion (e.g., potassium supplementation) after measurement—fundamentally impacts interpretation and limits causal inference.

Future research directions include: (1) conducting prospective interventional studies to evaluate whether correcting the Na^+^/K^+^ ratio improves patient prognosis; (2) developing combined predictive models integrating the Na^+^/K^+^ ratio with other biomarkers; and (3) employing multi-omics approaches to elucidate the molecular mechanisms by which the Na^+^/K^+^ ratio influences outcomes in cardiac arrest.

## 5 Conclusion

In conclusion, this study demonstrates a significant U-shaped relationship between the serum Na^+^/K^+^ ratio and ICU mortality in patients with cardiac arrest. Monitoring this ratio may aid in risk stratification and guide clinical decision-making.

## Data Availability

The original contributions presented in this study are included in this article/Supplementary material, further inquiries can be directed to the corresponding author.

## Footnotes

1
http://www.R-project.org
