# Supplementary material for: The association between the sodium-potassium ratio and ICU mortality in cardiac arrest patients: an analysis of the eICU database
Source: Front Med (Lausanne). 2026 Jun 5;13:1855533. doi: 10.3389/fmed.2026.1855533 (PMC13279623; doi:10.3389/fmed.2026.1855533)
Supplement: Supplementary file 1 [file Supplementary_file_1.docx]

1. **Table S1 Covariate screening table**

2、**Table S2. Comparison of naive vs. clustered bootstrap standard errors**

| **Variable** | **Model 1** | **Model 2** | **Notes** |
| --- | --- | --- | --- |
|  | *Naive / Clustered** | *Naive / Clustered** |  |
| T2 vs T1 | 0.72 (0.64–0.81) / 0.72 (0.65–0.81) | 0.81 (0.71–0.92) / 0.81 (0.73–0.90) | Ref: T1 |
| T3 vs T1 | 0.77 (0.68–0.85) / 0.77 (0.70–0.83) | 0.87 (0.76–0.99) / 0.87 (0.79–0.94) | Ref: T1 |

** Clustered bootstrap 95% CI, 300 hospital-level resamples. Model 1: age, sex, race, temperature, heart rate. Model 2: Model 1 + albumin, RDW, BUN, calcium, chloride, creatinine, hemoglobin, magnesium, WBC.*

**Interpretation:** Point estimates are identical between naive and clustered approaches. Clustered CIs are marginally wider but direction, magnitude, and significance remain unchanged (robust to clustering adjustment).
