# Supplementary material for: The association between the sodium-potassium ratio and ICU mortality in cardiac arrest patients: an analysis of the eICU database
Source: Front Med (Lausanne). 2026 Jun 5;13:1855533. doi: 10.3389/fmed.2026.1855533 (PMC13279623; doi:10.3389/fmed.2026.1855533)
Supplement: Supplementary file 2 [file Supplementary_file_2.docx]

1. **Figure S1 Proportional test**


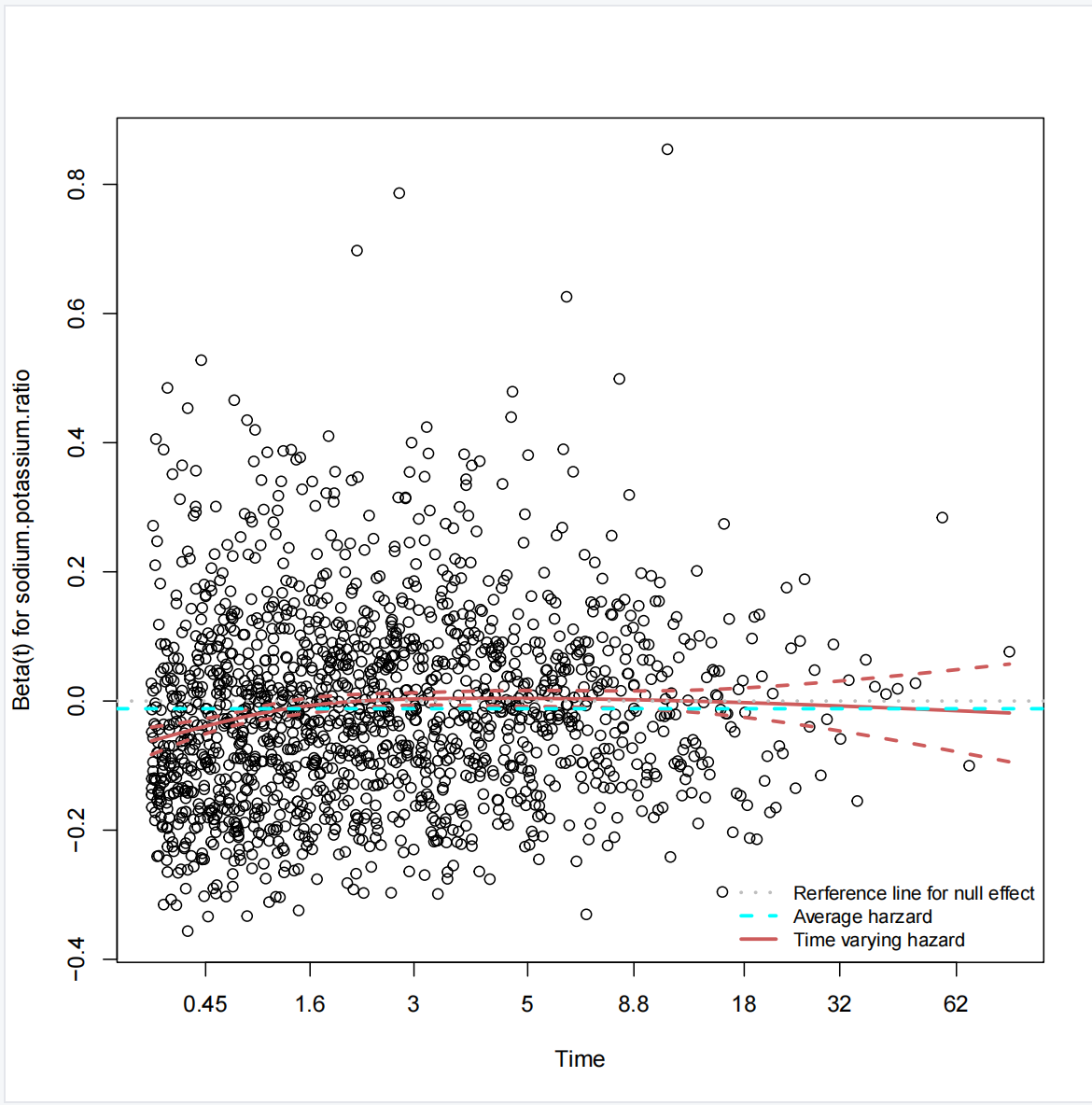


1. **Table S1 Proportional test**

1. **Table S2 hazard ratios (95% confidence intervals) of ICU mortality and different Sodium-potassium ratio in different models.**

| Outcomes | Group | Non-adjusted | | Model 1 | | Model 2 | |
| --- | --- | --- | --- | --- | --- | --- | --- |
|  |  | HR(95% CI) | *P* | HR(95% CI) | *P* | HR(95% CI) | *P* |
| ICU mortality | Sodium-potassium ratio.tertile1 | 1(Ref) |  | 1(Ref) |  | 1(Ref) |  |
|  | Sodium-potassium ratio.tertile2 | 0.72 (0.64~0.81) | <0.001 | 0.72 (0.64~0.81) | <0.001 | 0.81(0.71~0.92) | 0.001 |
|  | Sodium-potassium ratio.tertile3 | 0.8 (0.71~0.89) | <0.001 | 0.76(0.68~0.85) | <0.001 | 0.87 (0.76~0.99) | 0.03 |

Model 1 was adjusted for race, gender, temperature and heart rate.

Model 2 was additionally adjusted for albumin, RDW, BUN, calcium, chloride, creatinine, HGB, magnesium and WBC.
